# Assessing Confidence in Antidepressant Prescribing Amongst the Foundation Year Cohort

**DOI:** 10.1192/bjo.2025.10445

**Published:** 2025-06-20

**Authors:** Abigail Spiteri, Gareth Smith

**Affiliations:** NELFT, London, United Kingdom

## Abstract

**Aims:** The aim of this quality improvement project was to assess, and improve the confidence in antidepressant prescribing and management of common side effects amongst the Foundation Year (FY) trainees.

**Methods:** PDSA cycles involving a questionnaire sent out to 25 FY doctors in psychiatric jobs.

**Results:** A survey was sent out to the FY cohort currently doing a psychiatry job placement to assess their baseline confidence in antidepressant prescribing, which highlighted a lack of confidence. With regards to management of depression and anxiety, 25% felt unconfident, 50% felt neutral, and 25% felt somewhat confident. With regards to prescribing antidepressants, 75% felt neutral, and 25% felt somewhat confident. When it came to managing side effects of antidepressants, 25% felt very unconfident, 50% felt somewhat unconfident, and 25% felt somewhat confident.

A flowchart detailing indications for antidepressant prescribing and basic principles on swapping and titrating doses was distributed to help improve confidence. The group was reassessed with the same questionnaire to accurately identify any improvement.

When re-assessed, there was still a lack of confidence with regards to the management of side effects, with 63% feeling somewhat unconfident, 13% feeling neutral, and 25% feeling somewhat confident.

A presentation was prepared and delivered at the weekly teaching, which detailed the use of common antidepressants, their side effects, and the management of complications including serotonin syndrome.

The cohort was again re-assessed, showing an overall improvement in confidence in all aspects, with 80% feeling somewhat confident in management of depression and anxiety, and 100% feeling somewhat confident in prescribing antidepressants and management of side effects.

**Conclusion:** Positive outcomes included an overall improvement in confidence in prescribing antidepressants. Additionally, some participants found that the gaps in their knowledge were greatly reduced through these two cycles of information sharing.

Potential improvements to the study include using a larger cohort sampling outside of the FYs in psychiatry rotations to get a broader idea of the general FY cohort confidence. Furthermore, some participants still feel they have gaps in their knowledge, which could be addressed through more teaching sessions tackling individual cases in the future.

